# Was the Scanner Calibration Slide used for its intended purpose?

**DOI:** 10.1186/1471-2105-12-110

**Published:** 2011-04-21

**Authors:** Shannon Zhang, Yaping Zong

**Affiliations:** 1 Full Moon BioSystems, Inc., 754 North Pastoria Avenue, Sunnyvale, CA 94085, USA

## Abstract

In the article, *Scanner calibration revisited, BMC Bioinformatics 2010, 11:361*, Dr. Pozhitkov used the Scanner Calibration Slide, a key product of Full Moon BioSystems to generate data in his study of microarray scanner PMT response and proposed a mathematic model for PMT response [1]. In the end, the author concluded that "Full Moon BioSystems calibration slides are inadequate for performing calibration," and recommended "against using these slides." We found these conclusions are seriously flawed and misleading, and his recommendation against using the Scanner Calibration Slide was not properly supported.

## Introduction

To scanner users, the most important goal is to correctly quantify a feature on a microarray image and determine whether the signal intensity, an arbitrary reading from the scanner, is a real signal, which is directly affected by noise and other background variations [2]. The Scanner Calibration Slide was designed by Full Moon BioSystems to simulate real microarray chips, which are comprised of substrates, printing buffer and printed material. The users can easily assess their scanners in an environment that represents actual microarray experiments, without having to worry about the different sources of noises. The Scanner Calibration Slide allows users to establish a working curve using a simple dilution series of fluorescent material printed on the slide, to asses microarray scanner's general performance, and to evaluate and compare multiple scanner systems. It is an excellent reference tool. Full Moon BioSystems has been selling this product for more than eight years with an excellent scientific record [3-5]. It has been widely used as a quality assessment and evaluation tool by scanner manufacturers and scanner owners around the world [5-7].

In *Scanner calibration revisited, BMC Bioinformatics 2010, 11:361*, Dr. Pozhitkov used the Scanner Calibration Slide to generate a series of data in effort to analyze PMT response in microarray scanners [1]. However, he chose not to follow the product instructions in utilizing the data derived from the product. Therefore, his conclusion, "Full Moon BioSystems calibration slides are inadequate for performing calibration," and recommendation against "using these slides" are flawed and misleading.

## Analysis

Following product instructions is key to ensure that the product is used for the intended purpose. We must point out that Dr. Pozhitkov did not follow the product instructions in utilizing the data derived from the product. Instead he applied the data in a manner he deemed appropriate. In his study, using the data generated by the Scanner Calibration Slides, Dr. Pozhitkov established several working curves to analyze PMT response. However, none of them was established following the instructions in the User's Guide of the Scanner Calibration Slide, which is readily available online at http://www.fullmoonbiosystems.com. The product instructions specifically ask the users to use signal-to-noise ratio (SNR) to construct a working curve in analyzing and comparing scanners' key attributes and features. Instead, the author used the "pure signals," which are scanner readouts, directly affected by noises, PMT voltages, gains and other hardware settings. They vary from scanner to scanner. Therefore, they are not a good measurement of the scanner's attributes. On the other hand, signal-to-noise ratio is a standard metric used to analyze and compare performance and results from different scanners [2,8,9]. It determines how well a signal is differentiated from the noise of the system and qualifies the accuracy of a given signal measurement [2]. Certainly, Dr. Pozhitkov is free to use the data derived from this product in any manner he desires. However, it is unfair for Dr. Pozhitkov to conclude that the product should not be recommended in general and for its intended purpose when he chose not to follow the instructions and used the product for a different purpose. Furthermore, Dr. Pozhitkov claims in this article that "the autofluorescence of the Scanner Calibration Slide's buffer was responsible for the lower plateau" in the working curve he established. In fact, the plateau is related and affected by many factors including background fluorescence, which involves noises inherent to the scanner system, fluorescence from printing buffers and substrates, and other variations [9]. We do not dispute the fact that the printing buffer used on Scanner Calibration Slide has trace of fluorescence. In fact, all commonly used printing buffers in microarrays, for example, DMSO, SSC, phosphate buffer, produce some degree of fluorescence. The surface of the microarray chips produces fluorescence as well. That is why background correction is necessary when performing DNA or protein microarray analysis. Furthermore, all scanners have noises including dark current noise and shot noise, which are intrinsic to the scanner system [2]. The dark current noise is produced by the thermal emissions from the photosensor and leakage current through the dynodes of the photomultiplier tubes (PMT) or any other photon-detecting device. The shot noise is originated by the fundamental particle nature of light. Both types of noise are found in all optical measuring systems, and they both produce background and affect a microarray image [2]. The level of noise varies from system to system, and the noises produce background signals even when scanning is done without a glass slide. As signals approach background, quantitative accuracy diminishes. In general, the limit of detection is defined as the minimum detectable signal for which the signal-tonoise (SNR) is 3 [9]. When properly constructed, the plateau in the working curve established by the Scanner Calibration Slide indicates the scanner response has reached its limit of detection and can no longer discriminate the difference in fluorescent signals. Nonetheless, the author failed to address how any of these factors affects the plateau. Therefore, the author's claim that attributes the lower plateau solely to the autofluorescence in the printing buffer was not fully substantiated.

Lastly, Dr. Pozhitkov's analysis is contradictory and lacks proper support. He relied on the data derived from the Scanner Calibration Slide, but later concluded that the Slide should not be recommended. Further Dr. Pozhitkov did not use any other method to validate his proposed formula for PMT response, nor did he demonstrate by any other method or technique why the Scanner Calibration Slide is not adequate for its intended use. The results and conclusion from this kind of contradicting study cannot be trusted.

## Conclusion

In summary, we believe Dr. Pozhitkov failed to use the Scanner Calibration Slide for its intended purpose, and his recommendation against the product cannot be considered valid.

## Competing interests

Shannon Zhang and Yaping Zong are employed by Full Moon BioSystems, Inc.

## Authors' contributions

SZ and YZ helped to draft the manuscript. All authors read and approved the final manuscript.

## Response to: Was the Scanner Calibration Slide Used for Its Intended Purpose?

Alexander Pozhitkov,

Max Planck Institute for Evolutionary Biology, August-Thienemann-Str. 2, Ploen, 24306, Germany.

Alexander.pozhitkov@evolbio.mpg.de

## Response

The major argument of Drs. Zhang and Zong is that the research [1] was flawed because it used raw signal intensities instead of the SNR. It is a surprising notion because the research in question was a follow-up of Shi et al. [3], who also used the raw signal intensities. One would wonder why the respected opponents did not subject the latter study to a similar criticism... In fact, that work is of excellent quality and this is why it drew my attention to use the Calibration Slide for another study [11].

I have no doubts about the SNR being an important characteristic when the signal part of it is meaningful. It was discovered [1] that a large portion of the signal was due to the autofluorescence of the buffer. Drs. Zhang and Zong admitted that "the printing buffer used on Scanner Calibration Slide has trace of fluorescence". I believe this is an understatement: the signal intensity of the "buffer" spots of the Calibration Slide is several times higher than that of the glass part (images recorded on Agilent Scanner, 2010).

Drs. Zhang and Zong demonstrate quite a thorough knowledge on particle scattering and the nature of the dark current. We certainly "speak the same language", because most of my group's research is focused on the microarray physics [10-16]. Nevertheless, while the dark current and light scattering do produce some signal, the major contributor to the background is the buffer. As was mentioned above, the signal intensity of the "buffer" spots is several times higher than the glass.

With the understanding that the dilution series of the dyes on the Calibration Slide is compromised by the background of the buffer, one would wonder if the SNR measurements are meaningful. In fact, about half of the dilutions on the Scanner Calibration Slide (13 out of 27, [1]) do not significantly differ from one another due to the autofluorescence. Not only the SNR measurements are poor, the scanner response function is incorrect. At least my study [1] was able to theoretically predict the scanner behavior in the absence of the autofluorescence.

In conclusion, given the autofluorescence of the buffer, the SNR and any other measurements are seriously distorted and should not be used to characterize scanners until the issue of the autofluorescence is fixed. I hereby confirm the conclusions of the paper [1] and reiterate the recommendation against using the Fill Moon BioSystems Scanner Calibration Slide until the issue of the autofluorescence is resolved. I invite Drs. Zhang and Zong to conduct a joint study to improve the calibration slide, because it will be an invaluable tool for the microarray community.
